# Genome-wide association study of Senegalese sorghum seedlings responding to a Texas isolate of *Colletotrichum sublineola*

**DOI:** 10.1038/s41598-022-16844-6

**Published:** 2022-07-29

**Authors:** Ezekiel Ahn, Coumba Fall, Louis K. Prom, Clint Magill

**Affiliations:** 1 Department of Plant Pathology and Microbiology, Texas A&M University, College Station, TX 77843 USA; 2 Present Address: USDA-ARS Plant Science Research Unit, St. Paul, MN 55108 USA; 3 USDA-ARS Southern Plains Agricultural Research Center, College Station, TX 77845 USA

**Keywords:** Genetics, Microbiology, Plant sciences

## Abstract

*Colletotrichum sublineola* is a destructive fungal pathogen that causes anthracnose in sorghum. Senegalese sorghum germplasm is currently being considered as an option of sources for genetic resistance**.** In a recent study, Senegalese sorghum accessions were evaluated for response to a mixture of Texas isolates of *C. sublineola* at the 8-leaf stage in the greenhouse. As a comparison, 159 Senegalese sorghum accessions at the 1-leaf developmental stage were evaluated against a single Texas isolate of *C. sublineola* (FSP53) using an excised-leaf assay. A genome-wide association study (GWAS) was conducted based on the phenotypic data acquired to discover genetic variation associated with response to *C. sublineola* using 193,727 single nucleotide polymorphisms (SNPs) throughout the genome. Sorghum seedlings tended to be more resistant when compared with sorghum plants inoculated at the 8-leaf stage in the greenhouse in previous experiments. Based on the highest score evaluated in the 1-leaf developmental stage excised leaf assay for each accession, 16 accessions were labeled as susceptible. GWAS identified the SNP locus S01_72868925 that is associated with protein kinase domain // Leucine rich repeat N-terminal domain at a level of confidence that surpassed Bonferroni correction. Along with the SNP locus S01_72868925, other top SNP loci were also associated with genes that are known to play critical roles in plant defense or plant stress responses.

## Introduction

Sorghum [*Sorghum bicolor* (L.) Moench] is a multipurpose food crop which is ranked among the top five cereal crops in the world^1^. Like other important cereal crops, sorghum is consistently challenged by biotic and abiotic stresses that constrain overall productivity^2^. Sorghum anthracnose caused by *Colletotrichum sublineola* Henn. ex Sacc. & Trotter 1913 is an important disease of cultivated sorghum worldwide^3^. In recent years, genome-wide association studies (GWAS) have identified potentially important defense-related genes against *C. sublineola* in various sorghum germplasms^4–6^. As examples, genes that encode an F-box, a protein tyrosine kinase, a leucine rich repeat and a peroxidase were identified as top candidates for anthracnose resistance in the sorghum association panel (SAP) lines^4^. Similar studies identified genes whose products include motifs such as pentatricopeptide repeat and a leucine-rich repeat (LRR) as top candidates in the same SAP lines^5^.

In the sorghum mini core collection, genes that encode proteins with a zinc finger domain, an F-box domain, exodeoxyribonuclease VII, and ubiquitin-conjugating enzyme were listed as top candidate defense related genes^6^. Many germplasm lines from West and Central Africa where sorghum is cultivated in rainy and high humidity regions have been shown to be important sources of resistance genes to fungal diseases^7^. In a recent study, 163 Senegalese sorghum accessions were scored for responses to a mixture of eight Texas isolates (FSP2, FSP5, FSP7, FSP35, FSP36, FSP46, FSP50 and FSP53) of *C. sublineola*^8^. Plants of the Senegalese accessions were grown in a greenhouse, inoculated at the 8-leaf stage, and the subsequent GWAS analysis identified genes that encode leucine rich repeat // protein tyrosine kinase // leucine rich repeat N-terminal domain, selenium binding protein and zinc finger as genes highly associated with defense against Texas isolates of *C. sublineola*^8^. Sorghum seedlings contain the cyanogenic glycoside dhurrin, which may play a role in seedling protection^9^, and a phytoalexin pigment complex accumulates rapidly in sorghum seedlings when inoculated to *C. sublineola* (formerly known as *C. graminicola* (Ces.) G.W. Wils) ^9,10^. Therefore, compared to 8-leaf stage of sorghum, seedlings may show greater resistance against *C. sublineola* that can easily skew phenotypic data toward resistance. Previously however, comparisons between anthracnose inoculations made at juvenile and later growth stages of sorghum have not been studied in sorghum.

To compare the response of sorghum plants inoculated at the 8-leaf stage, 159 Senegalese sorghum accessions were evaluated at the 1-leaf stage for response to a Texas isolate of *C. sublineola* through an excised-leaf assay^11^. All 159 accessions were the same as those included in the previous study for greenhouse survey at the 8-leaf stage^8^ (insufficient seed was available for the other 4 lines). It is hypothesized that Senegalese sorghum accessions would respond differently when inoculated at 1-leaf seedling stage compared to conventional 8-leaf stage. Moreover, it is hypothesized that an excised-leaf assay^11^, originally designed to be used in sorghum at the 8-leaf stage, can be practical for earlier screening of sorghum seedlings. By applying GWAS analysis to disease response based on an excised-leaf assay on sorghum seedlings, it was also hypothesized that it is possible to identify defense related genes that are particularly important at seedling stage against anthracnose. As in the previous study, the disease score results were combined with a GWAS analysis that includes 193,727 single-nucleotide polymorphic (SNP) loci from a publicly available genotype-by-sequencing (GBS) dataset for the Senegalese accessions^8^. GWAS analysis was conducted by using TASSEL^12^ version 5.2.80 based on the highest score for disease ratings in each accession; candidate genes that might contribute to resistance or susceptibility to anthracnose in Senegalese sorghum seedlings are reported.

## Results

### Phenotypic variation

**Figure 1:**
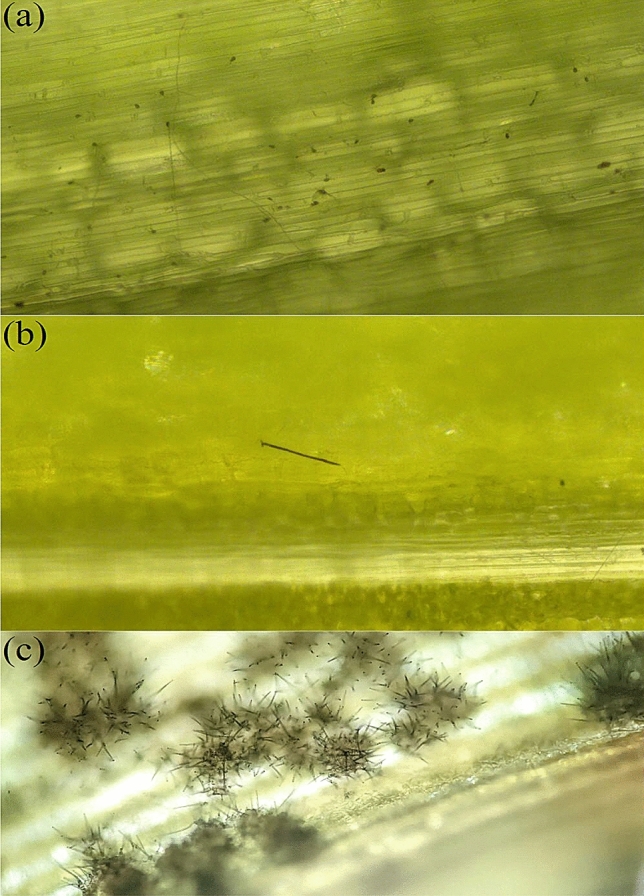
Acervuli formation was confirmed on leaf segments of Sorghum seedlings response to FSP53 at 96 h post-inoculation. (**a**) Visual representation of score 1. No visible infection was shown on a leaf segment of SC748-5 (resistant check) (**b**) Visual representation of score 2. Germ tube formation was confirmed on a leaf segment of SC748-5 (**c**) Acervuli formation was confirmed on a leaf segment of PI514300. Although not prevalent, FSP53 successfully formed acervuli on Senegalese seedlings. Note: The images of (**a**) and (**b**) were used for visual representation of score 1 and 2. The images are not associated with this study as it captured responses of leaf segments of SC748-5 at 8-leaf stage to FSP53 at 96 h post-inoculation from a previous unpublished work.

Senegalese seedlings overall showed strong resistance to FSP53; average scores based on all 9 1st leaves for all accessions were between 1 and 1.78 including resistant and susceptible checks. However, the accessions showed considerable variation based on the highest scores among 9 tested 1st leaves. Excluding resistant and susceptible checks, 82 accessions scored 1, and 61 accessions scored 2. Six accessions (PI514284, PI514298, PI514299, PI514372, PI514455 and PI514459) scored 3, and 3 accessions (PI514427, PI514449 and PI514474) scored 4. Lastly, 7 accessions (PI514293, PI514300, PI514448, PI514457, PI514467, PI514473 and PI514478) had at least one 1st leaf with a score of 5. The susceptible checks BTx623, PI609251, and TAM428 scored 3, 3, and 5 respectively. SC748-5, the resistant check, scored 2. As the majority of accessions using the excised leaf assay scored 1 or 2 at 1-leaf stage (seedlings exhibit only 1st leaf and flag leaf), juvenile plants of these Senegalese accessions can be assumed as generally resistant to the Texas isolate FSP53 of *C. sublineola* (Fig. 1a,b are representing score 1 & 2). Yet, 16 cultivars showed clear sign of infection (Fig. 1c).

In a previous study, the same sorghum accessions inoculated at the 8-leaf stage in a greenhouse rather than using excised-leaf assays were generally resistant to a mixture of Texas isolates^8^, but many different outcomes were shown when compared to the results from this study (Table 1).

**Table 1:** Reaction of the Senegalese accessions inoculated with a *C. sublineola* isolate from Texas (FSP53) at 1-leaf stage through excised-leaf assay compared with reaction of the identical accessions at the 8-leaf stage inoculated through greenhouse spray inoculation with a mixture of Texas isolates of *C. sublineola* in 2019^8^. In sum, 84 accessions showed different results in the two studies. Accessions not listed in Table 1 were resistant in both studies^8^.

Accession	1-leaf excised-leaf assay	8-leaf greenhouse inoculation	Accession	1-leaf excised-leaf assay	8-leaf greenhouse inoculation
PI 514,279	R	S	PI 514,377	R	S
PI 514,283	R	S	PI 514,378	R	S
PI 514,284	S	S	PI 514,380	R	S
PI 514,288	R	S	PI 514,388	R	S
PI 514,289	R	S	PI 514,391	R	S
PI 514,293	S	S	PI 514,392	R	S
PI 514,296	R	S	PI 514,393	R	S
PI 514,298	S	R	PI 514,394	R	S
PI 514,299	S	R	PI 514,395	R	S
PI 514,300	S	R	PI 514,396	R	S
PI 514,304	R	S	PI 514,399	R	S
PI 514,305	R	S	PI 514,400	R	S
PI 514,306	R	S	PI 514,404	R	S
PI 514,308	R	S	PI 514,405	R	S
PI 514,309	R	S	PI 514,409	R	S
PI 514,310	R	S	PI 514,411	R	S
PI 514,311	R	S	PI 514,412	R	S
PI 514,312	R	S	PI 514,414	R	S
PI 514,313	R	S	PI 514,417	R	S
PI 514,319	R	S	PI 514,419	R	S
PI 514,323	R	S	PI 514,420	R	S
PI 514,324	R	S	PI 514,423	R	S
PI 514,325	R	S	PI 514,424	R	S
PI 514,326	R	S	PI 514,427	S	R
PI 514,332	R	S	PI 514,430	R	S
PI 514,336	R	S	PI 514,432	R	S
PI 514,338	R	S	PI 514,437	R	S
PI 514,339	R	S	PI 514,438	R	S
PI 514,341	R	S	PI 514,444	R	S
PI 514,343	R	S	PI 514,446	R	S
PI 514,346	R	S	PI 514,448	S	R
PI 514,347	R	S	PI 514,449	S	R
PI 514,349	R	S	PI 514,455	S	R
PI 514,351	R	S	PI 514,457	S	S
PI 514,352	R	S	PI 514,459	S	R
PI 514,353	R	S	PI 514,462	R	S
PI 514,355	R	S	PI 514,463	R	S
PI 514,356	R	S	PI 514,464	R	S
PI 514,360	R	S	PI 514,465	R	S
PI 514,361	R	S	PI 514,467	S	R
PI 514,367	R	S	PI 514,473	S	S
PI 514,368	R	S	PI 514,474	S	R
PI 514,372	S	R	PI 514,478	S	R
PI 514,374	R	S	PI 609,251(−)	S	S

### Genome-wide association study

**Figure 2:**
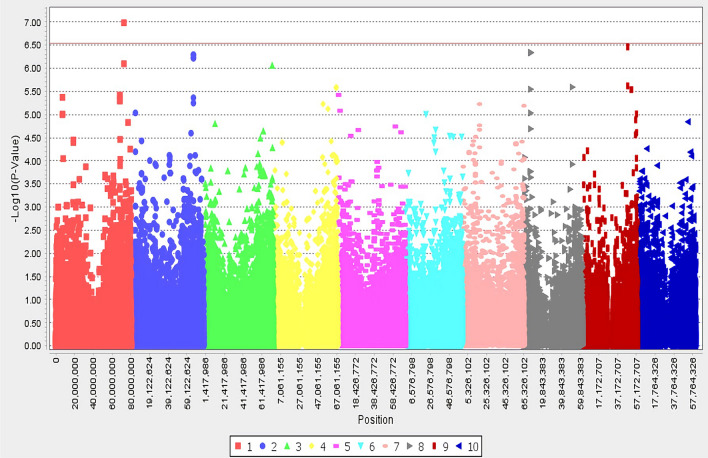
The genome-wide association for response to anthracnose in Senegalese sorghum seedlings. Manhattan plot showing locations of SNP-detected QTLs associated with response to FSP53 on the ten chromosomes of *Sorghum bicolor* at 1-leaf stage. Bonferroni correction≈0.00000039 after filtering out SNPs with greater than 20% unknown alleles with minor allele frequency (MAF) below 5%. The red line indicates Bonferroni correction.

The SNP locus S01_72868925 is the only SNP that passed the Bonferroni threshold, but Table 2 listed 3 high confidence candidates close to the associated SNPs (Fig. 2 and Table 2).

**Table 2:** Annotated genes nearest to the most significant SNPs associated with anthracnose to 1-leaf stage seedlings. The distance in base pairs to the nearest genes and *P*-value are listed. Two major alleles were calculated to verify significant differences for scores based on Student’s t-test, and SNPs failed to show differences were not considered as top candidates. * = passed Bonferroni correction (Bonferroni correction≈0.00000039).

Chr	Location	Candidate gene and function	Base pairs	SNP base %	TASSEL *P-*value	Average score/SNP
1	72,868,925 and 1 more within 1 bp	Sobic.001G451800 Protein kinase domain // Leucine rich repeat N-terminal domain	0	G:80.1% T:19.9%	0.000000104*	G:1.55 T:2.13
8	7,370,058 and 2 more within 3 bp	Sobic.008G065800 Poly (ADP-ribose) polymerase, catalytic domain	16,202	A:21.9% C:78.1%	0.00000046	A:1.91 C:1.54
9	51,943,886	Sobic.009G162500 K05280-flavonoid 3'-monooxygenase	0	C:81.5 T:18.5%	0.0000029	C:1.59 T:2.03

When mapped back to the published sorghum genome, top candidates SNPs that passed or nearly passed Bonferroni correction were nearest to genes that have previously reported roles in biotic or abiotic resistance/stress responses.

## Discussion

Sorghum seedlings are known to be more resistant compared to later growth stages. It has been assumed this may be because they contain the preformed cyanogenic glycoside dhurrin, which may play a role in seedling protection^9^. Similarly, cultivars of Johnsongrass [*Sorghum halepense* (L.) Pers.], a wild relative of sorghum, were reported to be more resistant in young plants compared to fully grown plants when inoculated to a sorghum isolate of *C. sublineola*^13^. As Table 1 depicts, the results followed the previously reported patterns when compared to 8-leaf stage inoculated sorghum plants^8^. Majority of accessions were resistant in seedling stage, but they become more susceptible at the 8-leaf stage. In contrast, 11 cultivars showed completely opposite results; susceptible seedlings were detected, but acervuli formation was not observable in 8-leaf stage plants in the previous study (PI514298, PI514299, PI514300, PI514372, PI514427, PI514448, PI514449, PI514455, PI514459, PI514474 and PI514478)^8^. Both of these changes infer different resistance mechanisms operate in juvenile versus older plants. If dhurrin is inhibitory, these plants may simply be those with naturally low levels. The single isolate used in these studies was selected due to its pathogenicity on BTx635 at both stages of growth whereas a mix of isolates known to represent different pathotypes was used in in the previous tests. Nevertheless, a recent study showed that following inoculation with single or a mixture of two isolates of *C. sublineola*, had minimal to no effect, in that infection rates were more characteristic for the virulent isolate in mixed inoculum^14^. The use of the single isolate may contribute the decrease in number of high scores, but as FSP53 strain has a strong and broad virulence pattern to sorghum cultivars^5,11^, the effect is assumed to be minimal. The results of screening sorghum to *C. sublineola* can also be altered by environmental effects. For example, screening results in Texas and Puerto Rico differed in a study^8^. Similarly, SAP lines responded differently to anthracnose based on inoculum and field locations^4^. PI514284, PI514293 and PI514473 were susceptible at both seedling and 8-leaf stage^8^. It is speculated that change of phytoalexin levels may vary from seedling to 8-leaf stage in individual sorghum accessions.

The SNP locus S01_72868925 is located in protein kinase domain // Leucine rich repeat N-terminal domain (Sobic.001G451800). LRRs are a feature of nearly all cloned resistance genes and are widely known for roles in plant host defense^8^. Although not the same SNP, GWAS analysis based on identical accessions after inoculation at the 8-leaf stage showed the SNP locus S06_60609133 as the top candidate SNP, and the SNP locus S06_60609133 tagged leucine rich repeat/protein tyrosine kinase (Sobic.006G274866)^8^. In other studies, sorghum mini core collection and SAP lines, regarding anthracnose and head smut, leucine rich repeat containing proteins were commonly listed as top candidate genes^4–6^, but chromosomal locations differed in each study.

The SNP locus S08_7370058 is 16202 bp away from a poly (ADP-ribose) polymerase (PARP), catalytic domain. PARP domains have also been implicated as factors in stress responses of plants^15^. Transgenic plants with reduced PARP levels have broad-spectrum stress-resistant phenotypes^16^.

The SNP locus S09_51943886 is located within the gene coding for flavonoid 3'-monooxygenase. Chalcone synthase (CHS) is a key enzyme of the flavonoid/isoflavonoid biosynthesis pathway^17^. CHS expression causes accumulation of flavonoid and isoflavonoid phytoalexins and is involved in the salicylic acid defense pathway^17^.

As sorghum seedlings typically contain the preformed cyanogenic glycoside dhurrin^9^, it was expected to see majority of 1st leaves to be scored as 1. Sorghum pathologists often label an accession as susceptible when even a single sorghum plant among a number exposed to inoculum can be infected. An example is head smut caused by *Sporisorium reilianum* (Kühn) Langdon & Fullerton. Similarly, a GWAS analysis based on the highest score of the 1st leaves based on the excised-leaf assay in each accession identified candidate genes associated with SNP loci supported by strong statistical power. As sorghum responses differed at the 1-leaf stage versus the 8-leaf stage, it is not surprising to identify novel SNP loci, including the SNP locus Sobic.001G451800 that are associated with seedling defense, but it will be essential to explore the response more deeply in the future. It is quite possible that different SNPs are highly associated with sorghum defense in seedling stage only or throughout the whole growth stages. The candidate genes identified in this study can be tested for further analysis such as real-time quantitative reverse transcription PCR (Real-time qRT-PCR) or RNA sequencing analysis (RNA-Seq) to confirm that different genes are highly expressed in seedling stages. Gene editing technology can also be applied to verify the defensive roles of the candidate genes. Lastly, level of cyanogenic glycosides can be measured at seedling and 8-leaf stages to find associations with phenotypes and the identified genes.

In sum, this study explored sorghum seedling responses to *C. sublineola* with an excised-leaf assay and verified that the excised-leaf assay can be useful to study seedlings, but compared to 8-leaf stage inoculation methods, a greater number of seedlings should be screened to determine susceptibility in each accession as the results can easily be skewed.

However, the excised-leaf assay applied to 1-leaf stage seedlings is expected to promote studies associated with sorghum seedlings and *C. sublineola* interactions; it can be rapidly conducted within laboratory setting.

## Methods

### Sorghum lines

The 159 accessions (listed in Supplemental Information for the accessions and raw scores to *C. sublineola*) were obtained from the USDA-ARS Plant Genetic Resources Conservation Unit. BTx623, TAM428, and PI609251 (susceptible) and SC748-5 (resistant) were used as positive and negative controls^8,18^.

### Excised-leaf assay and disease evaluations

Plug flats with 40 Square Cells (L x W x H≈ 5 cm × 5 cm × 7 cm for each cell) (The HC Companies, Twinsburg, OH) were filled with Metro Mix 200 (Sun Gro Horticulture, Agawam, MA), and sorghum accessions were grown at 23 °C with 65% humidity under LED light for approximately 12 h a day. When seedlings reached at l-leaf stage, an excised-leaf assay described by Prom et al.^11^ and Ahn et al.^19^ was applied. Prom et al.^11^ developed the assay to screen 8-leaf stage sorghum plants, but in this study, 1-leaf stage seedlings were used. In brief, a Texas *C. sublineola* isolate FSP53 was inoculated onto half strength Potato Dextrose Agar (PDA) plate and grown in an incubator for 2 weeks. FSP53 is one of the most virulent isolates based on the response of susceptible checks BTx623, TAM428, and RTx430 in field evaluation^5^, and the identical Senegalese sorghum accessions’ response to a mixture of Texas isolates including FSP53 were screened at the 8-leaf stage in a greenhouse recently^8^. Approximately 50 ml of sterile water was added to the plate, and a sterile spatula was used to scrape and remove colony growth, and the suspension mixture was screened through four layers of cheesecloth to obtain conidia^19^. Conidia concentrations were adjusted to a f 10^6^ conidia/ml^19^. For the excised-leaf assay, whole leaves of each cultivar were placed, adaxial side up, on a half strength PDA plate, and 5 μl of the spore suspension was inoculated on the leaf blade^19^. In each trial, three 1st leaves were inoculated for each accession, and 3 trials of the excised-leaf assay were conducted (Total number of inoculated 1st leaves = 9 throughout 3 trials in each accession). Excised 1st leaves were inspected under an Olympus BX60 microscope (Olympus Co., Shinjuku, Tokyo, Japan) with 10 × magnification at 96 h post-inoculation, and scored for seedling responses to *C. sublineola* were recorded by using a 1–5 scale where 1 = fully resistant without visible fungal infection; 2 = fungal germ tube formed; 3 = fungal bed formed with some imperfectly formed acervuli; 4 = 1–5 acervuli perfectly formed and 5 = more than 5 acervuli perfectly formed^19^. Among 9 observed 1st leaves, the highest score was used to determine the symptom types to be categorized into two reaction types: Ratings 1 or 2 as resistant; ratings 3–5 as susceptible^19^. All raw scores are listed in Supplemental Information.

### GWAS Analysis

SNP data from a recent study^8^ were used. Originally, SNP data were extracted from an integrated sorghum SNPs dataset based on sorghum reference genome version 3.1.1 and originally genotyped using GBS^20–23^, and missing data were imputed using Beagle 4.1^24^.

TASSEL^12^ version 5.2.80 was used to conduct a mixed linear model (MLM) association analysis based on the highest score for disease ratings in each accession. False associations were reduced by removing SNPs with greater than 20% unknown alleles. Furthermore, SNPs with minor allele frequency (MAF) below 5% were removed as well. SNPs contributing to seedling responses to *C. sublineola* were tracked to the specific chromosome location by using the sorghum genome sequence, version 3.1.1 available at the JGI Phytozome 13 website^25^. To verify statistical significance, the mean disease rating score for all Senegalese accessions with either of the two prevalent bases was determined and verified to differ significantly (*P* < 0.05) based on Student’s T-test by using JMP Pro 15 (SAS Institute, Cary, NC, USA)^5^. Any top candidate SNP with (*P* ≥ 0.05) were removed from the results reported in Table 2.

## Supplementary Information

Supplementary Information.

## Data Availability

The raw phenotypic data is available as a Supplemental Information. The SNP dataset used is originally from a study deposited to Dryad Data Repository (https://doi.org/10.5061/dryad.63h8fd4, database/repository name: An integrated genotyping-by-sequencing polymorphism map for over 10,000 sorghum genotypes)^21^.
